# Threatening Sounds as an Alternative Mitigation Method to Deter Elephant: A Study in Elephant Conservation Centre, Kuala Gandah, Pahang, Malaysia

**DOI:** 10.21315/tlsr2025.36.1.3

**Published:** 2025-03-30

**Authors:** Norasmah Basari, Mohamad Firdaus Abd Sata, Nor Zalipah Mohamed, Fatin Nursyafiqah Zamri, Haslina Samsudin, Mohd Faizol Shamrie, Muhammad Adhwa Ikhwan Latif, Siti Norzahira Yazid, Aisyah Humairah Mohd Zaidi

**Affiliations:** 1 Faculty of Science and Marine Environment, Universiti Malaysia Terengganu, 21030 Kuala Nerus, Terengganu, Malaysia; 2 TDM Berhad, Aras 5, Bangunan UMNO Terengganu, Lot 3224, Jalan Masjid Abidin, 20100 Kuala Terengganu, Terengganu, Malaysia

**Keywords:** Animal Behaviour, Beehive-fence, Elephant Encroachment, Human-elephant Conflict, Oil Palm Plantation, Threatening-sound, Kelakuan Haiwan, Pagar Sarang Lebah, Pencerobohan Gajah, Konflik Manusia-Gajah, Ladang Kelapa Sawit, Bunyi Ancaman

## Abstract

Human-elephant conflict is a common issue in the agricultural sector, often resulting in crop damage. This study aimed to evaluate the effectiveness of threatening vocalisation playbacks as a mitigation method to deter elephant encroachment into agricultural areas. The study was conducted at the National Elephant Conservation Centre (NECC) in Kuala Gandah, Pahang, Malaysia, involving two male and five female elephants. Five soundtracks were played to observe the elephants’ responses: the sound of a buzzing bee, a tiger roar, an elephant rumble, rain (control) and nocturnal jungle sounds (control). The elephants’ behaviours were recorded during and after exposure to each soundtrack. The results showed that the elephants responded most strongly to the tiger roar (33%), followed by the buzzing bee sound (23%), while the elephant rumble elicited the fewest responses (8%). The tiger roar and buzzing bee sounds also resulted in the longest halt times, with the elephants stopping and standing still, particularly the older group (*p* < 0.05). Male and female elephants exhibited similar responses to the sound playbacks (*p* > 0.05). This study suggests that playback of threatening vocalisations could serve as an additional mitigation strategy to deter elephants from encroaching on agricultural sites, such as oil palm plantations.

## Abstract (fr)

**Abstrak:**

Konflik manusia-gajah adalah isu lazim dalam sektor pertanian, selalunya mengakibatkan kerosakan tanaman. Kajian ini bertujuan untuk menilai keberkesanan rakaman bunyi ancaman sebagai kaedah mitigasi untuk menghalang pencerobohan gajah ke kawasan pertanian. Kajian ini telah dijalankan di Pusat Konservasi Gajah Kebangsaan (PKGK) di Kuala Gandah, Pahang, Malaysia, melibatkan dua ekor gajah jantan dan lima gajah betina. Lima rakaman bunyi dimainkan untuk memerhatikan tindak balas gajah: bunyi dengungan lebah, ngauman harimau, deruman gajah, bunyi hujan (kawalan) dan bunyi hutan pada waktu malam (kawalan). Tingkah laku gajah dirakam semasa dan selepas pendedahan kepada setiap rakaman bunyi. Keputusan menunjukkan bahawa gajah bertindak balas paling ketara terhadap ngauman harimau (33%), diikuti dengan bunyi dengungan lebah (23%), manakala deruman gajah menunjukkan tindak balas paling rendah (8%). Ngauman harimau dan dengungan lebah juga menyebabkan gajah berdiri kaku dan diam lebih lama, terutamanya bagi kumpulan gajah yang lebih tua (*p* < 0.05). Gajah jantan dan betina mempamerkan tindak balas yang sama terhadap rakaman bunyi yang dimainkan (*p* > 0.05). Kajian ini mencadangkan bahawa rakaman bunyi ancaman boleh menjadi strategi mitigasi tambahan untuk menghalang gajah daripada menceroboh tapak pertanian, seperti ladang kelapa sawit.

**Highlights**

- Elephants respond most strongly to the sound of a tiger’s roar (33%), followed by buzzing bees (23%).
- The older group of elephants (over 40 years) showed significantly longer halt and alert durations compared to the younger group (under 40 years).
- Male and female elephants exhibited similar behaviour in response to threatening sounds (*p* > 0.05).

## INTRODUCTION

Human-elephant conflicts (HECs) were recorded as early as 1960 in Peninsular Malaysia, when elephants were often killed as a solution (Department of Wildlife and National Parks Malaysia 2016). These conflicts happened due to factors such as habitat fragmentation, forest destruction and land use changes (Sukumar 2003) which have resulted in the elimination of most lowland habitats available for elephant roaming (Shaffer *et al*. 2019). The loss of large forest areas in Peninsular Malaysia has significantly contributed to the country’s economy in the agricultural sector (Gillis 1988). In the 1990s to 2000s, approximately 56% of deforested areas were converted into oil palm plantations in Peninsular Malaysia (Shevade *et al*. 2017). Therefore, the elephants are attracted to agricultural areas as new feeding grounds, particularly oil palm and other plant crops (Ahmad Zafir & Magintan 2016). This has led to substantial losses for plantation owners. According to the Department of Wildlife and National Parks Malaysia (2016), approximately 68% of HECs in Peninsular Malaysia were due to crop raiding by elephants from 2011 to 2015, with 933 reported cases in 2015.

Integrated Pest Management (IPM) is a strategy used to manage problem animals and situations in human-wildlife management (Rashwin & Sanjeeth 2023). Therefore, HEC mitigation in oil palm plantations used two approaches from IPM, which are exclusion techniques and frightening devices. Exclusion techniques involve using on the use of high-voltage electrified fences to prevent elephant encroachment on oil palm plantations (Food and Agriculture Organizations of the United Nations 2008). However, this method is generally costly and considered to be a long-term investment, particularly for commercial perennial crops (Sukumar 2003). In addition, mitigation techniques like loud noises and burning fires are more cost-effective, but their effectiveness was short-lived, as elephants quickly became habituated to them (de la Torre *et al*. 2021; Fernando *et al*. 2008; Perera 2009). Hence, this phenomenon needs an effective and inexpensive method to mitigate the elephant’s encroachment on oil palm plantations. Beehive fences are currently used as a method to deter elephants from encroaching on oil palm plantations (Ndlovu *et al*. 2016; Abdul Malek *et al*. 2023).

A study conducted by Dror *et al*. (2020) in northern Thailand by using captive Asian elephants and beehive fences reported that encroachment on crop fields by Asian elephants (*Elephas maximus*) may be deterred by using African bees (*Apis mellifera*) or Asian bees (*Apis cerana*). However, they stated that their findings were different from those of King *et al*. (2018) because the bees exhibited low aggression levels and the elephants were unresponsive to the bees. King *et al*. (2018) conducted their study in Sri Lanka by involving 120 wild African elephants (*Loxondota Africana*) that are exposed to the playback sound of disturbed hives of Asian bees (*Apis cerana)*. Thuppil and Coss (2016) tested the playback of felid growls and human shouts on Asian elephants (*Elephas maximus*) in southern India, finding deterrence rates of 57.1% for human shouts, 72.7% for leopard growls and 90% for tiger growls.

Elephants exhibit aggressive behaviours when threatened. The sound of buzzing bees can alert elephants, causing them to retreat to avoid stings in sensitive areas like ears, trunks and eyes (King *et al*. 2007; Vollrath & Douglas-Hamilton 2002). Trunk-touching behaviour among the elephants during bee sound playback was interpreted as a nervous behaviour by seeking reassurance among them (Plotnik & de Waal 2014). Headshaking and dusting behaviours would knock bees away and flee from the area quickly to lower the risk of being stung (King *et al*. 2010). In addition, the elephants vocalise aggressively when exposed to leopard growls but retreat silently upon hearing tiger growls (Thuppil & Coss 2013). The elephants tended to linger in the vicinity to investigate the area prior to retreating by vocalising behaviour after hearing leopard growls (Thuppil & Coss 2013). However, playback sound’s long-term effectiveness is still unknown, as there is a possibility for animals to get habituated to the threats (Ndlovu *et al*. 2016).

No study has been performed on elephant behaviour towards various threatening sounds in Malaysia despite the high HEC incidence. It is unclear if elephants will react similarly to those in other countries, highlighting the need for local research to improve future mitigation methods. Hence, this study was conducted to determine how the elephants in Malaysia respond to threatening vocalisation sound playbacks and whether this method can improve conflict mitigation measures in the country.

## METHODS

### Study Site

This experiment was conducted from 7 August 2019 until 20 September 2019. This research was conducted at the National Elephant Conservation Centre (NECC), Kuala Gandah, Pahang, Malaysia. The NECC is situated approximately 100 km east of the capital city of Kuala Lumpur. NECC was an elephant’s sanctuary covering nearly 5.8 ha of the Krau Wildlife Reserve in Temerloh, Pahang, established in 1923 to protect wildlife species in the area (N 3° 35′32.28″, E 102°8′34.15″).

### Experimental Design

**Figure 1:**
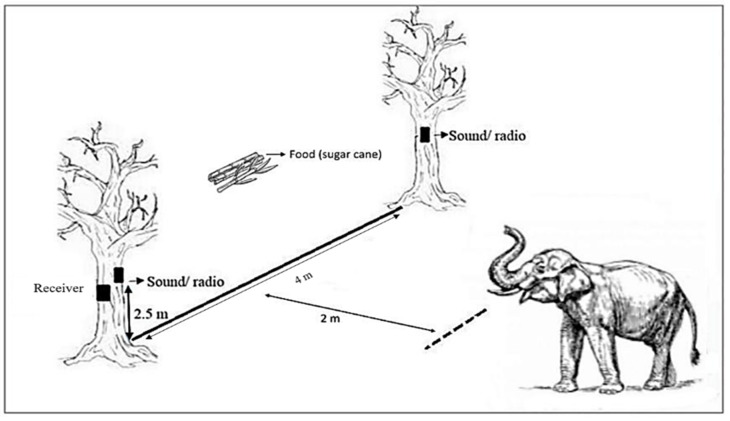

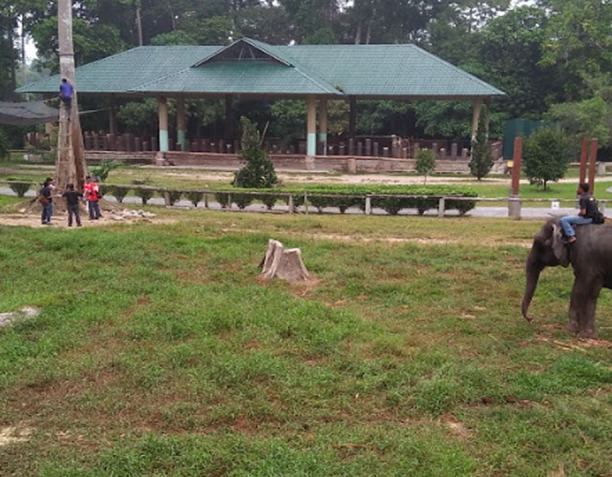

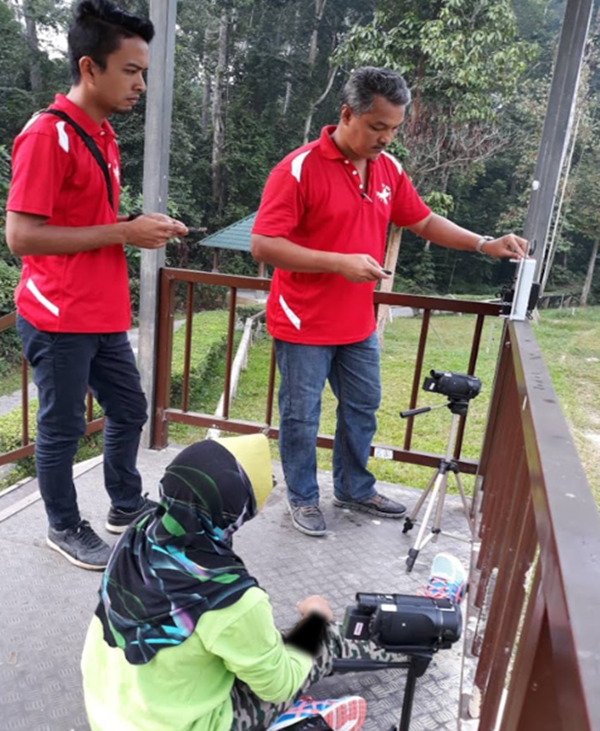

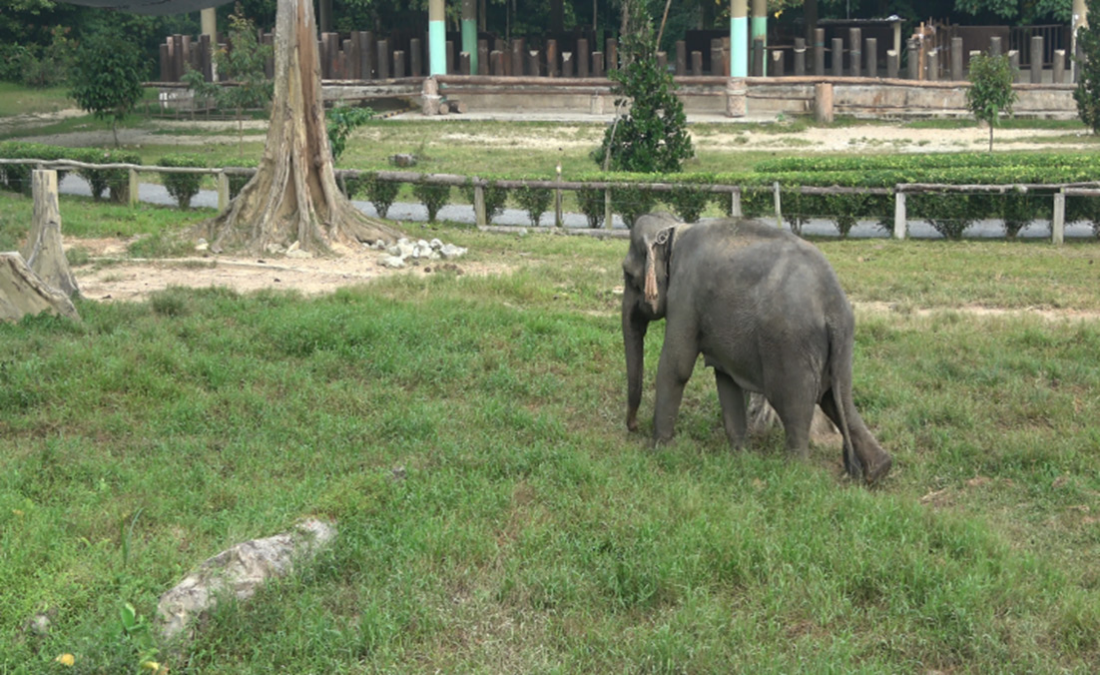
The experimental set-up. (a) a sketch of an actual set-up of the experimental area; (b) radios were hung on the trees as shown in (a); (c) the controller box used by the experimenters; (d) an example of the experiment trial where an elephant was allowed to roam inside the experiment area alone. A specific sound was played when the elephant stepped or passed the dotted line as shown in (a).

This experiment was conducted in a specific area or field covering around 8,093.71 m^2^ in the NECC. The perimeter of this field is surrounded by an electric fence (Fig. 1).

Seven healthy elephants were used in this study as permitted by the NECC management, consisting of two males, namely Alam (22 years old) and Lasah (21 years old), and five females, namely Kasturi (44 years old), Indah (23 years old), Kala (49 years old), Sanom (14 years old) and Mentopian (45 years old). These elephants originated from different parts of the forests in Peninsular Malaysia. These elephants were rescued from various parts of the forests in Peninsular Malaysia by the Department of Wildlife and National Parks (PERHILITAN). They were involved in incidents such as losing their herd or human-elephant conflict situations, leading to their relocation to the NECC for care and rehabilitation.

Five different sounds were used during the experiment, consisting of three threatening sounds and two white noises as controls. The three threatening sounds were the tiger roar, elephant rumble and buzzing bee sound, while the white noises (control sounds) were the rain and nocturnal jungle sounds. The sounds used in this experiment were downloaded from the free sound effects website, pixabay.com (https://pixabay.com/). The experiment began at 7:00 a.m. until 9:00 a.m., before any visitor could enter the NECC. Three radios were used to play the sound. Two radios were hung on two trees, with a distance of 4 m between the trees. The hanging radios were 2.5 m above ground, and a receiver box was attached to one of the trees near the radio (Fig. 1). These radios were connected via Bluetooth to a receiver and the main controller box held by the experimenter. A dotted line was set up using small broken sticks (so as not to draw attention from the elephant on trial) two meters before the feeding line (Fig. 1) that connected to the two trees. A bunch of sugar cane was placed in front of the feeding line as bait. The feeding line separates the bait area and the area where the experimenter starts to switch on the controller box using a remote control and play the respective sound (marked with a dotted line in Fig. 1a).

During the experiment, only one elephant was allowed to enter the experiment area to facilitate data recording and control the sound playbacks. The elephant was left roaming around the area, and its behaviour was observed. Once the elephant passed or stepped on the dotted line area, a specific trial sound was played at 80 dB intensity level for 5 min, and the elephant’s response to the sound was recorded using digital cameras for 10 min. The elephant responses were observed based on the elephant behaviour guide, as suggested by Olson (2024). Each elephant was tested once for each sound (seven replicates per sound, *N* = 35).

## STATISTICAL ANALYSIS

The data of elephant behaviour versus various threatening sounds of male and female elephants was analysed using a chi-square test for goodness of fit and the data of the elephant halt duration versus various types of sounds were analysed using a chi-square test for relatedness. The difference in the halt duration between older elephant (over 40 years old) versus younger elephant (below 40 years old) was conducted using Mann-Whitney U test. All analysis were performed using SPSS version 24 (IBM Corp., USA).

## ETHICAL NOTE

Only healthy elephants, as permitted by the NECC management, were used in this study. The mahout (the elephant keeper) of each elephant was present at the experimental area throughout the experiment to control the elephants’ behaviour in case they misbehaved. The experimenters and the mahout remained outside the electric fence while the experiments were being conducted. No elephant nor the experimenters were injured during this experiment. This experiment was approved by PERHILITAN (license number: T-00106-15-18).

## RESULTS

**Figure 2:**
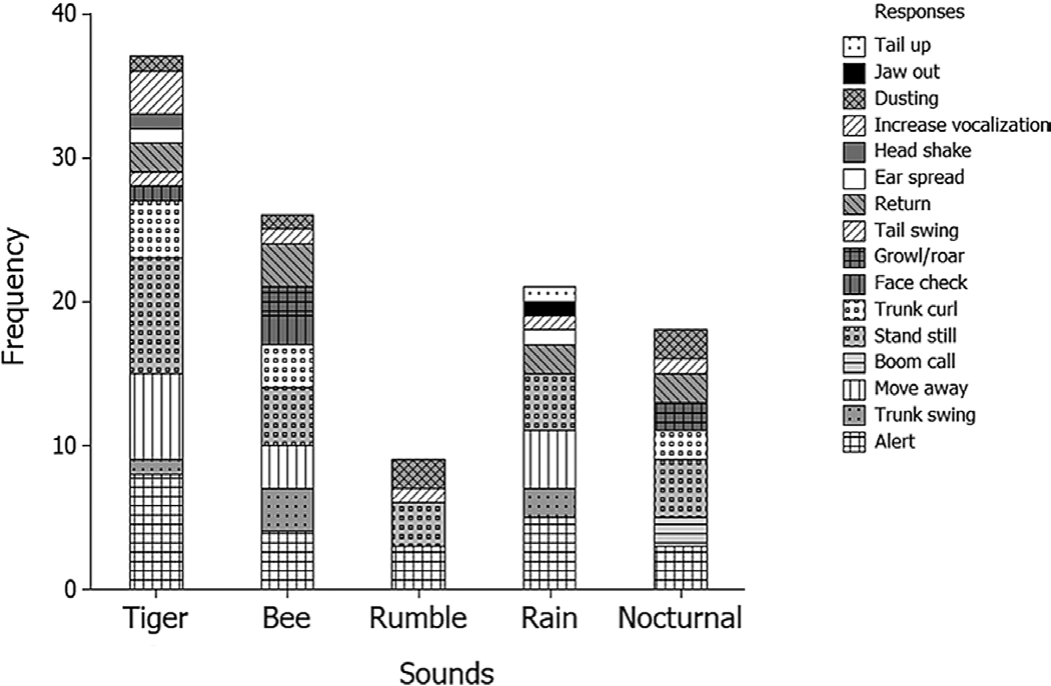
The frequency of responses shown by the elephants after each playback sound. The tiger sound caused the elephant to show various types of behaviour and highest frequency followed by the buzzing bee sound.

**Figure 3:**
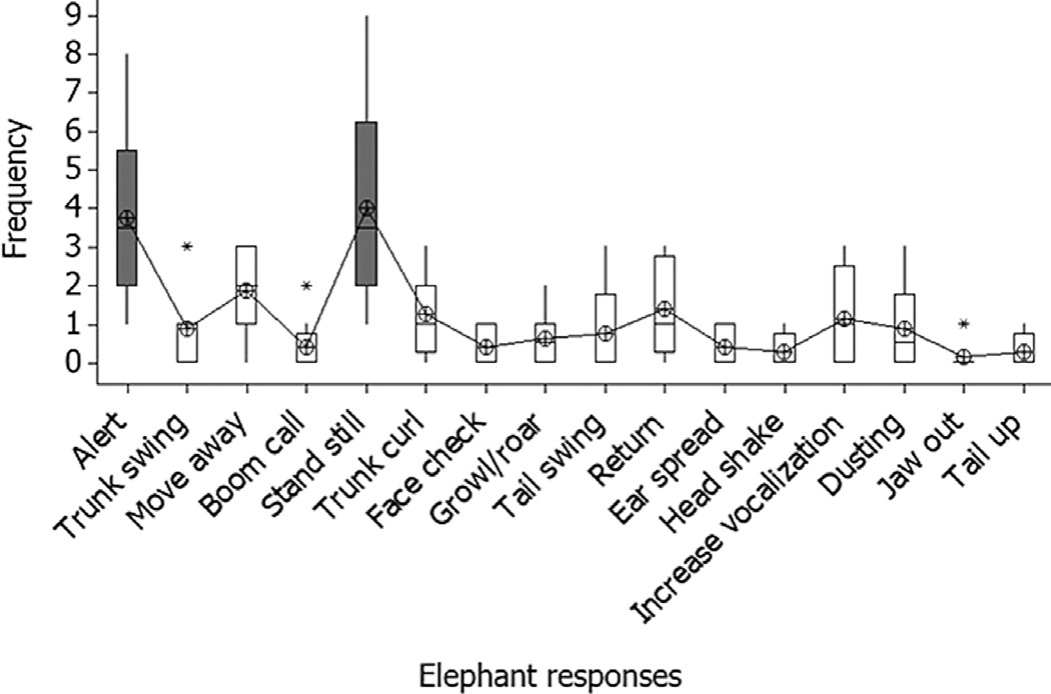
The frequency of overall responses shown by elephants towards all sounds.

**Figure 4:**
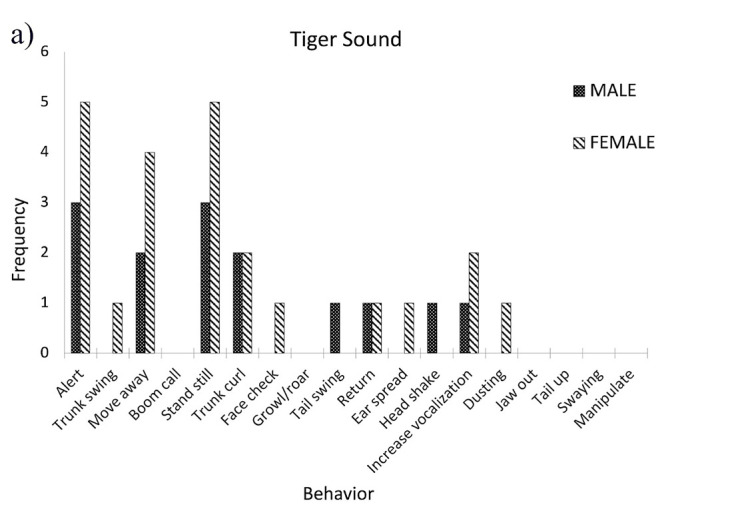

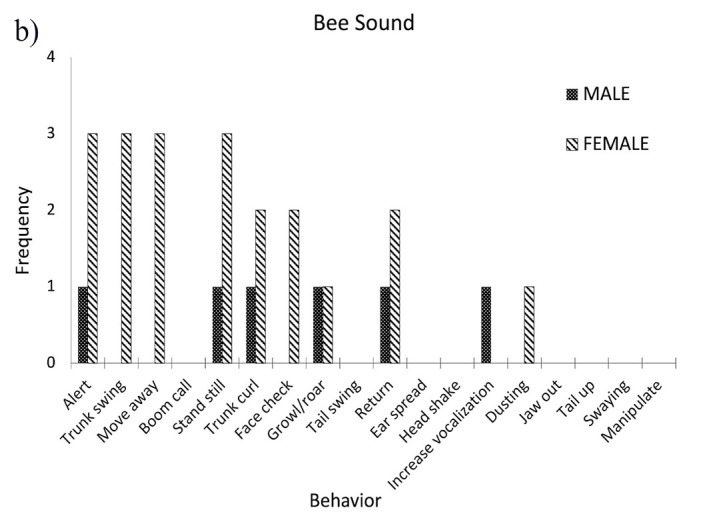

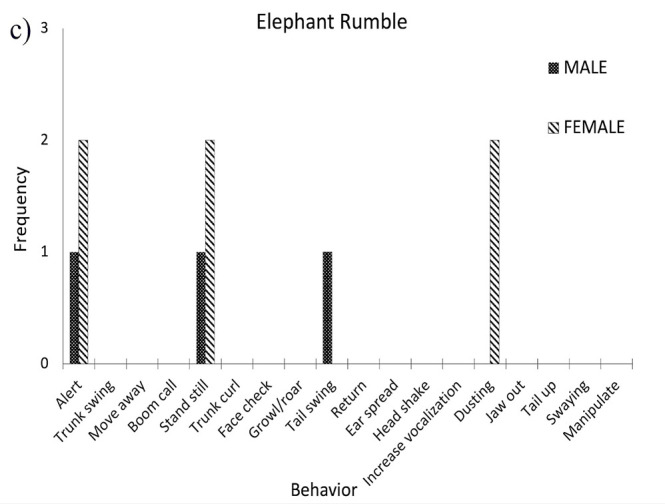

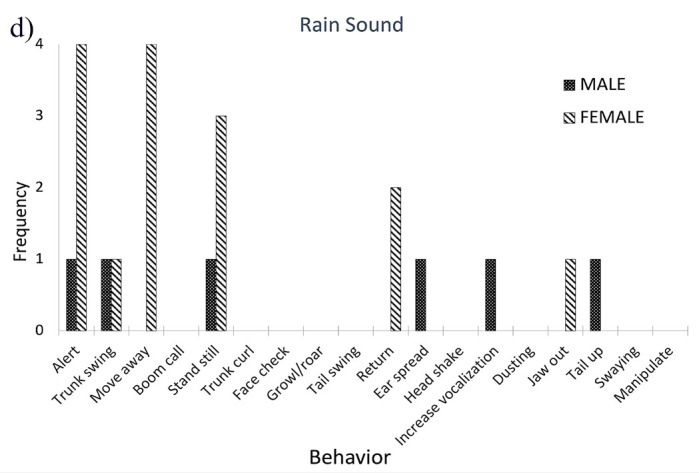

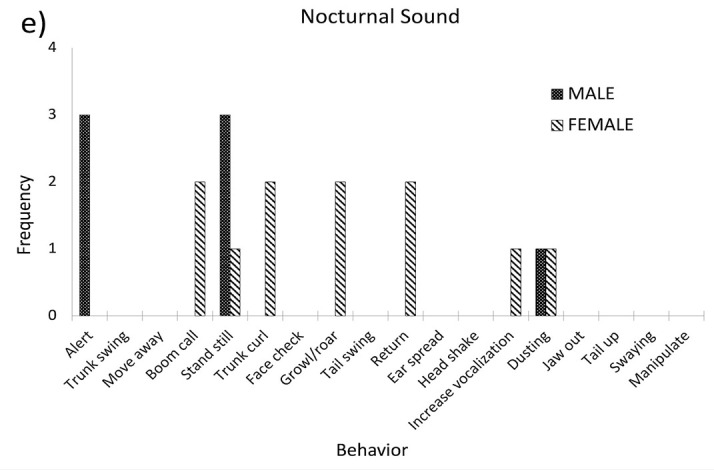
The frequency of male and female elephant response towards different types of sounds.

The elephants responded most strongly to the tiger sound (33%), followed by the bee sound (23%), while the elephant rumble sound elicited the fewest responses (8%) (refer Fig. 2). Fig. 3 illustrates the frequency of behaviours exhibited by male and female elephants in response to specific sound playbacks. The majority of responses shown by male and female elephants when the tiger sounds were played were that they stood still and became alert toward their surroundings. Upon hearing bee sounds, male elephants stood still, exhibited alertness, demonstrated trunk curling, growled and retreated to the starting point. Meanwhile, female elephants stood still, became alert, swung their trunks and subsequently retreated from the sound sources. Interestingly, the elephants also showed alertness when the rain sounds were played. They stood still, became alert, swung their trunk, spread their ears, raised their tail and increased vocalisation. Female elephants were seen to be more alert and later retreated from the sound playback. Lastly, when the nocturnal sounds were played, the male elephants stood still and later induced alertness, while female elephants tended to make boom calls, curled their trunks, growled and retreated from the sound source. Overall, there is a significant difference in the total frequency of behaviours shown by the elephants after certain sounds were played. Most of the elephants showed alert and standstill behaviour when a certain sound was played (χ^2^ = 103.82, df = 15, *p* < 0.001; Fig. 3). However, the frequency of responses shown by male and female elephants was not significantly different (χ^2^ = 19.39, df = 1, *p* = 0.415; Fig. 4).

**Figure 5:**
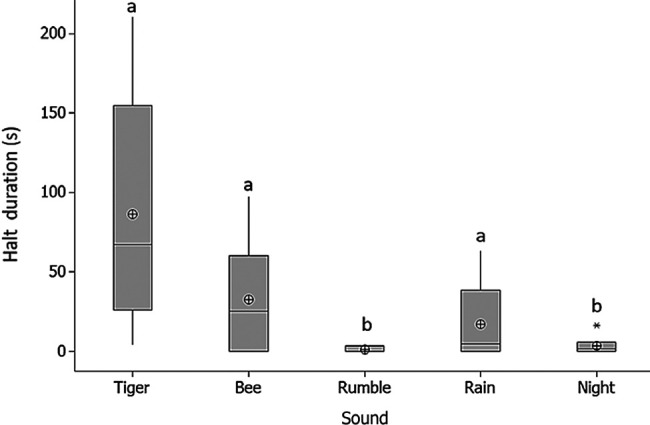
The boxplot showing the halt duration of elephants after each of the soundtrack playback.

**Figure 6:**
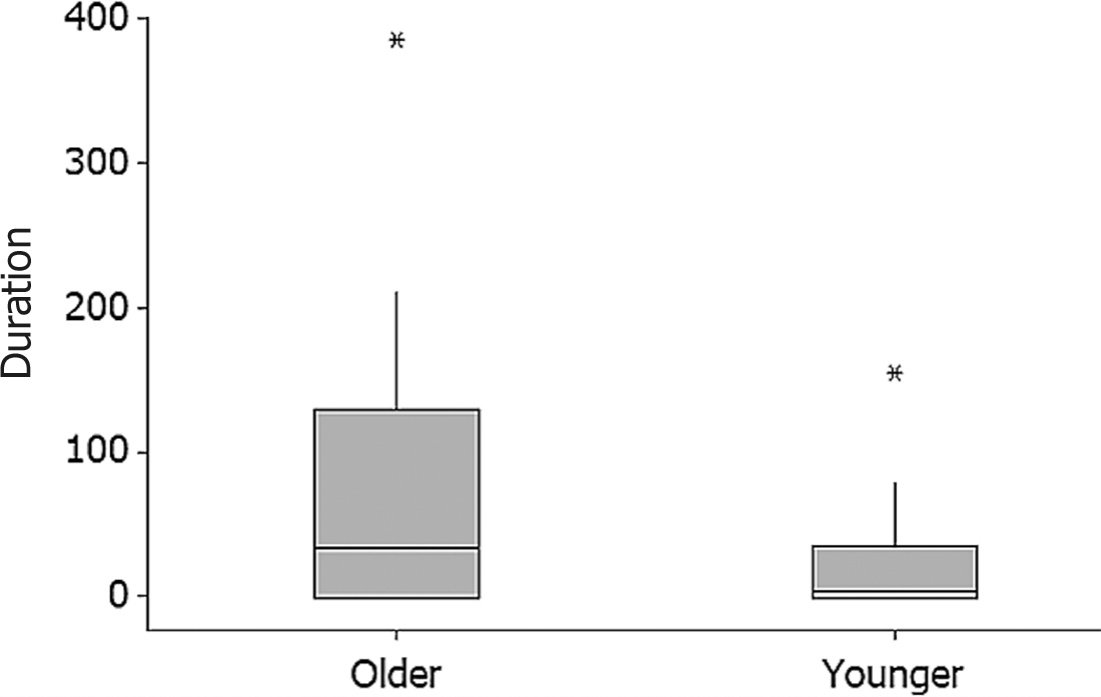
The boxplot showing the halt duration of elephants by age groups. Older elephants defined by elephants aged over 40 years old while younger elephants represented by elephants aged below 40 years old.

The halt duration, where elephants paused and stood still, was significantly longer when tiger, bee and rain sounds were played compared to elephant rumble and nocturnal sounds (χ^2^ = 1038.63, df = 4, *p* < 0.001; Fig. 5). The tiger sound and the bee sound recorded fewer elephants crossing the feeding line to feed on the sugar cane after five minutes of sound playbacks. Our results also showed that older elephant (aged over 40 years old) significantly had longer alert and halt time upon hearing the threatening sound compared to younger group of elephants (below 40 years old) (U = 22.00, Z = −2.714, *p* = 0.005; Fig. 6).

## DISCUSSION

In this study, the frequency of the elephant’s response was recorded using five types of soundtracks, which were buzzing bee sound, tiger sound, elephant rumble, rain sound and nocturnal jungle sound.

The tiger roar and buzzing bee sound elicited the strongest threat response from the elephants, inducing behaviours such as alertness, moving away and increased vocalisation, compared to elephant rumble and nocturnal sounds. The highest frequency of response observed across all the five sounds was the tiger roar and the bee sound. During our experiment, we clearly observed that some elephants were threatened by the tiger roar and buzzing bee sounds. When the tiger and bee sounds were played, the elephants usually stood still for quite a long time before retreating from the feeding line. Our observations align with Thuppil and Coss (2016), who found that tiger and lion growls effectively deter elephants from crop areas. Elephants retreat quietly from potential threats like tigers but increase vocalisations with aggressive sounds such as trumpets to announce their presence. Their study also reported that elephants did not stay longer in the area when they heard a tiger growl sound. They even showed alert behaviour and investigative behaviour (Thuppil & Coss 2013).

In current study, elephants primarily displayed alert behaviour by standing still when a soundtrack was played. The duration of alertness was significantly longer during the tiger roar and buzzing bee sounds. The elephants growled and increased vocalisation when all sounds were played except for the rumble sound. Likely, these elephants had previously encountered tiger threats in the jungle before being brought to the NECC. This experience likely made them more alert upon hearing the tiger roar sound playbacks. It is also intriguing to find that older elephants exhibit greater alertness than younger ones. This observation aligns with established patterns in animal behaviour, where older individuals, having accumulated more experience, are generally better at identifying and assessing threats than their younger counterparts (McComb *et al*. 2011; Weissing 2011).

Previous studies have shown that when an African elephant is exposed to disturbed honeybee sounds, the elephants exhibit behaviour that appears to act as a defence against bees (McComb *et al*. 2001; Langbauer *et al*. 1991). Since elephants have a long and highly social memory, negative experiences within groups can lead to better and longer-term adjustments through social facilitation (Hinde 1966). Head shakes and dust will keep the bees away and escape the area quickly, reducing the risk of being stung. As elephants move away from the sound source, they produce a sound echo during and after the bee stimulation. Hence, the elephant is very sensitive to the bee sound. They also flip their ears to prevent being stung in the sensitive area. These disturbances may be expressions of moderate emotional intensity (Rendall 2003) or may serve as contact calls that coordinate group movements (Poole *et al*. 1988; Leighty *et al*. 2008) or as alarm calls to further elephants (Langbauer 2000; Poole *et al*. 1988). McComb *et al*. (2001) stated that it is possible that such calls are used in social facilitation to educate inexperienced and vulnerable youth on the same dangerous threats.

Interestingly, our findings also showed that the elephants had a longer halt duration when the rain sound was played. This was unexpected, as the rain sound was a control experiment and hypothesised to elicit no significant behaviour. The significantly longer halt duration during rain sounds is probably due to the elephant that may have stopped and waited for the rain to occur, given that this animal is known to enjoy rain by playing in the dirt during rain. For elephant rumble and nocturnal sounds, the elephants did not display behaviours indicating that they felt threatened. Therefore, most of them crossed the feeding line and fed on sugarcane even while the playback sounds were ongoing.

Overall, this study suggests that playback of threatening vocalisations, specifically tiger roars and buzzing bee sounds, could effectively deter elephants from entering agricultural sites like oil palm plantations. This method can also protect living spaces, especially houses near forest borders, where there is a high risk of wildlife encroachment, including elephants. Placing a device that produces threatening sounds at the perimeter of these houses could reduce the risk of human-elephant conflict. Additionally, we recommend using other types of threatening sounds, such as human shouts, and changing the sounds periodically. Elephants are intelligent and can become habituated to a specific stimulus if it is presented for too long.

## CONCLUSION

This study highlights the potential use of threatening sounds, specifically tiger roars and buzzing bee sounds, to effectively manage human-elephant conflict. Both sounds elicited the strongest threat responses, such as stopped and paused for a long time and moved away from the source. Interestingly, older elephants demonstrated greater sensitivity, likely due to their accumulated experience in identifying dangers. The unexpected prolonged halt during rain sounds suggests a natural inclination toward enjoying rain rather than perceiving it as a threat. Conversely, elephant rumbles and nocturnal sounds triggered minimal reactions, with elephants continuing to feed despite playback. These findings highlight the need for safe and flexible sound-based methods to protect agricultural areas as well as houses near forest borders.
